# Tranexamic acid in local infiltration analgesia cocktail for pain and swelling after total knee arthroplasty: a randomized controlled trial

**DOI:** 10.1186/s42836-026-00385-8

**Published:** 2026-04-13

**Authors:** Qiuru Wang, Dongmei Zhao, Changjun Chen, Ting Ma, Pengde Kang

**Affiliations:** 1 Department of Orthopedic Surgery, West China Hospital, Sichuan University, 37# Wainan Guoxue Road, Chengdu, 610041 China; 2 Sports Medicine Center&Department of Orthopedics and Orthopedic Research Institute, West China Hospital, Sichuan University, 363# Furong Avenue, Chengdu, 610041 China; 3 Department of Orthopedic Surgery, Shandong Key Laboratory of Rheumatic Disease and Translational Medicine, The First Affiliated Hospital of Shandong First Medical University (Shandong Provincial Qianfoshan Hospital), Jinan, 250000 China; 4 Anesthesia and Surgery Center, West China Hospital, Sichuan University, 37# Wainan Guoxue Road, Chengdu, 610041 China

**Keywords:** Total Knee Arthroplasty, Local Infiltration Analgesia, Analgesic Cocktail, Tranexamic Acid, Pain, Enhanced Recovery after Surgery

## Abstract

**Background:**

Peri-articular local infiltration analgesia (LIA) is a cornerstone technique in multimodal pain management during the peri-operative period of total knee arthroplasty (TKA). Tranexamic acid (TXA), an antifibrinolytic agent, is widely used to reduce post-operative bleeding in TKA. However, no study has systematically evaluated the impact of adding TXA as an adjuvant to the LIA cocktail on post-operative pain and swelling following TKA. The purpose of this study is to investigate the efficacy and convenience of incorporating TXA into the analgesic cocktail for alleviating pain and swelling after TKA.

**Methods:**

In this double-blind, randomized controlled trial, 100 patients undergoing TKA were allocated to either a TXA group or a control group. The TXA group received LIA with an analgesic cocktail consisting of ropivacaine, epinephrine, dexamethasone, and TXA, while the control group received an identical cocktail without TXA. The primary outcome was the pain score at rest at 24 h post-operatively. Secondary outcomes comprised pain scores at other time points, post-operative morphine consumption for rescue analgesia, time to first rescue analgesia, knee swelling rate, decrease in hemoglobin level, range of motion (ROM) of the knee, and incidence of complications.

**Results:**

The TXA group demonstrated significantly lower VAS pain scores at rest at 24 h post-operatively (3.5 ± 0.6 vs. 4.0 ± 0.7, *p* = 0.001, and a markedly smaller decline in hemoglobin levels (26.2 ± 7.1 g/L vs. 33.5 ± 7.5 g/L, *p* < 0.001. Knee swelling rates were also significantly reduced in the TXA group. However, the absolute difference in VAS scores did not exceed the reported minimal clinically important difference (MCID) for pain in TKA (typically 1.0 point). No significant differences were observed between the two groups in post-operative morphine consumption, time to first rescue analgesia, knee ROM, or complication rates.

**Conclusion:**

The addition of TXA to a commonly used LIA cocktail led to a statistically significant reduction in early post-operative pain and swelling, along with decreased blood loss, which may offer added convenience by streamlining the workflow. However, the reduction in pain did not meet the MCID, indicating limited clinical relevance in terms of analgesia. Future studies are needed to optimize the analgesic efficacy of TXA-containing cocktails.

**Trial registration:**

Chinese Clinical Trial Registry, ChiCTR2400086985. Registered 16 July 2024.

**Supplementary Information:**

The online version contains supplementary material available at 10.1186/s42836-026-00385-8.

## Introduction

Total knee arthroplasty (TKA) has become an effective treatment for end-stage knee diseases, significantly alleviating pain and improving functional outcomes in patients [1, 2]. Over the past two decades, substantial progress has been made in the peri-operative management of joint arthroplasty within enhanced recovery after surgery (ERAS) protocols. However, effective management of post-operative pain, swelling, and blood loss remains a major clinical challenge for both orthopedic surgeons and anesthesiologists [3, 4]. TKA is recognized as one of the most painful procedures in surgery [5–7]. It has been reported that over 60% of patients experience severe pain following TKA [5, 6]. post-operative pain leads to multiple adverse effects and complications, including significantly impaired sleep and quality of life, as well as increased risks of deep vein thrombosis, pulmonary embolism, surgical site infection, chronic pain development, and opioid dependence. These complications can hinder early mobilization, delay rehabilitation, and prolong hospital stay [8–10]. Similarly, significant post-operative knee swelling and blood loss are common and can exacerbate pain, impede early physiotherapy, increase transfusion rates, and negatively impact recovery [11, 12].

Multimodal analgesia is a strategy that combines medications with different mechanisms of action and non-pharmacological techniques to synergistically target pain pathways, thereby enhancing analgesic efficacy while reducing the side effects associated with single-agent therapy [13, 14]. This approach has become the clinical standard for peri-operative pain management in TKA. peri-articular local infiltration analgesia (LIA) serves as a cornerstone technique within multimodal analgesia protocols for TKA. LIA specifically targets peripheral sensory nerve endings through multiple injections of a cocktail containing local anesthetics combined with various adjuvants into the surgical site [15]. Currently, no standardized formulation exists for the analgesic cocktail used in LIA. Typical cocktail compositions consist of long-acting local anesthetics such as ropivacaine or bupivacaine as the primary agents, supplemented with adjuvants that commonly include opioids, corticosteroids, and epinephrine [16–18]. An ideal LIA adjuvant would not only enhance analgesia but also help address other peri-operative challenges like swelling and bleeding.

Tranexamic acid (TXA), an antifibrinolytic agent, is extensively used in TKA. When administered intravenously, it has demonstrated efficacy in reducing post-operative blood loss, hemoglobin decline, and transfusion requirements [19, 20]. The primary safety concern with TXA is the theoretical risk of thromboembolic events; however, a large body of evidence, including meta-analyses of randomized trials, has not shown an increased risk in patients undergoing orthopedic surgery [19, 20]. Recent studies have shown that local administration of TXA—either into the intra-articular space or peri-articular tissues—can achieve hemostatic effects comparable to intravenous administration with minimal systemic absorption, further mitigating potential systemic side effects [21, 22]. Moreover, combined intravenous and local TXA application has been reported to further enhance hemostatic outcomes compared with either route alone [23]. Several investigators have also documented that locally administered TXA can mitigate knee swelling after TKA [24, 25]. We hypothesized that incorporating TXA as an adjuvant into the LIA cocktail would alleviate post-operative swelling and pain by reducing local blood loss, while simultaneously providing local hemostasis and obviating the need for separate TXA injections, thereby simplifying the surgical workflow.

Based on this hypothesis, and given that no prospective randomized controlled trial (RCT) has specifically evaluated the analgesic efficacy of incorporating TXA into the LIA cocktail, this study aimed to systematically assess the efficacy and convenience of a TXA-containing analgesic cocktail on post-operative pain, swelling, and blood loss following TKA.

## Methods

This study was designed as a double-blind, RCT and received approval from the Clinical Trials and Biomedical Ethics Committee of our institution. Written informed consent was obtained from all participants prior to enrollment. This study was prospectively registered on the Chinese Clinical Trial Registry (Registration Number: ChiCTR2400086985). While the registry initially listed multiple primary outcomes, the final protocol was refined to designate the VAS pain score at rest at 24 h post-operatively as the sole primary outcome, as it is a validated and commonly used primary endpoint in peri-operative analgesic studies for joint arthroplasty [26, 27]. All other initially registered outcomes were analyzed as secondary outcomes. Artificial Intelligence (AI) was not used in the research and manuscript development. This study was conducted and is reported in accordance with the Transparency in Artificial Intelligence Narratives (TITAN) Guidelines 2025 [28]. This work has been reported in line with Consolidated Standards of Reporting Trials (CONSORT) Guidelines [29].

### Patient recruitment

The patients were recruited from August 2024 to June 2025. We consecutively enrolled patients meeting the following criteria: (1) Aged 18–80 years; (2) Scheduled for unilateral primary TKA for knee osteoarthritis; (3) Admitted to our institution between August 2024 and June 2025; (4) Having an American Society of Anesthesiologists (ASA) physical status classification of I to III.

Key exclusion criteria were summarized as follows: (1) significant systemic comorbidities (e.g., severe cardiopulmonary, hepatic, renal dysfunction, or uncontrolled diabetes); (2) conditions affecting the surgical site or outcomes (e.g., prior open knee surgery, severe deformity, neuromuscular disorders, or active infection); (3) specific contraindications to study medications (e.g., history of thromboembolic events, known hypercoagulable states, or allergies); and (4) factors impairing study participation (e.g., cognitive barriers, opioid dependence, or concurrent trial enrollment). The complete, detailed list of inclusion and exclusion criteria is provided in the Supplementary Materials.

### Randomization and blinding

A computer-generated simple (1:1) randomization sequence was produced using the RAND function in Microsoft Excel (Microsoft, Redmond, WA, USA) to allocate patients into two groups (TXA or Control). No stratification factors (e.g., age, gender) were applied. Investigator 1, who remained blinded to both group assignments and the overall study design, utilized this sequence to prepare sequentially numbered, individually sealed, opaque envelopes (not lined with aluminum foil) for each participant. On the morning of surgery, investigator 2 opened the envelopes in sequential order and assigned patients to their respective groups based on the enclosed allocation codes.

Prior to the procedure, investigator 2 notified the anesthesiologist of the group assignments. The corresponding analgesic cocktail was prepared in advance by the anesthesiologist in the central pharmacy. Following the induction of general anesthesia, the prepared cocktail was delivered to the operating room. The surgeon then performed LIA during the surgery. Throughout the trial, the surgeon, outcome assessor (Investigator 3), and the statistician (Investigator 4) were all blinded to group allocations to minimize bias.

### Surgery and peri-operative management

Preemptive analgesia was administered on the day before surgery, consisting of oral celecoxib (200 mg) and pregabalin (150 mg) given twice daily. All patients underwent standard general anesthesia. Thirty minutes prior to anesthesia induction, a senior anesthesiologist performed an adductor canal block using a previously described technique [30]. Surgery was conducted under general anesthesia via a midline skin incision and medial parapatellar approach, with all procedures involving cemented prostheses and performed by a single senior surgeon. A pneumatic tourniquet set at 240 mmHg was applied from incision until wound closure, and no surgical drains were used.

LIA was performed intra-operatively by the surgeon. The control group received a cocktail containing 0.2% ropivacaine, 2.0 μg/mL epinephrine, and 0.1 mg/mL dexamethasone. The TXA group received the same cocktail supplemented with 10 mg/mL TXA. Apart from the cocktail composition, both groups were treated identically. Before prosthesis implantation, 20 mL of the cocktail was injected into the posterior capsule, and another 20 mL was infiltrated around the medial and lateral collateral ligaments. After implantation, 20 mL was administered into the quadriceps and retinacular tissues, and 40 mL was injected into the adipose and subcutaneous layers. Intravenous TXA (15 mg/kg) was given before tourniquet release to minimize blood loss. For post-operative pain and nausea prophylaxis, intravenous sufentanil (5 μg), dexamethasone (10 mg), and tropisetron (5 mg) were administered 20 min before surgery concluded.

Post-operative pain was managed with twice-daily oral celecoxib (200 mg) and pregabalin (150 mg). If patients reported intolerable pain during hospitalization and the outcome assessor confirmed a visual analogue scale (VAS) score at rest ≥ 4 or a VAS score during motion ≥ 6, subcutaneous morphine hydrochloride (10 mg) was administered as rescue analgesia. Additional intravenous TXA (15 mg/kg) was administered at 3 and 6 h after surgery.

### Pre-operative education and standardized post-operative rehabilitation

To ensure uniformity and minimize confounding, all patients received identical pre-operative education and followed a standardized post-operative rehabilitation protocol, which was an integral part of our institutional ERAS pathway. pre-operatively, the nursing team instructed patients on the techniques for key post-operative exercises. post-operatively, the rehabilitation protocol was initiated upon the patient’s return to the ward and included the following interventions, applied consistently to both groups: cryotherapy was applied to the incision site; intermittent pneumatic compression was used after lower limb venous ultrasonography. On the first post-operative day, physiotherapeutic modalities (infrared lamp and ultrasonic therapy) were administered. Furthermore, the nursing and rehabilitation team guided patients through a structured exercise regimen at specific time points. This regimen included ankle pump exercises, straight leg raises, and active knee flexion training. On the first post-operative day, patients also began standing, transfer, and ambulation training under supervision.

### Outcomes

The primary outcome was the VAS pain score at rest at 24 h post-operatively. The VAS was selected for its validation as a standard measure for acute pain, its ability to assess clinical relevance through established thresholds (e.g., the minimal clinically important difference), its sensitivity to change in the early post-operative period, and its widespread use enabling direct comparison with prior TKA analgesia studies. Secondary outcomes comprised pain scores at additional time points, post-operative morphine hydrochloride consumption for rescue analgesia, time to first rescue analgesia, knee swelling rate, post-operative hemoglobin decline, knee range of motion (ROM), and incidence of complications. Since follow-up extended until 72 h after surgery—by which time most patients met discharge criteria—length of hospital stay was excluded from the outcome measures.

post-operative pain was evaluated using a VAS ranging from 0 (no pain) to 10 (worst imaginable pain) [31]. Assessments were performed both at rest and during active knee flexion (motion) at 3, 6, 12, 24, 48, and 72 h after surgery.

Knee swelling rate was calculated as the percentage increase in lower limb circumference relative to the pre-operative baseline: [(post-operative circumference − pre-operative circumference)/pre-operative circumference] × 100% [11]. To standardize measurements and minimize observer bias, a single blinded assessor performed all evaluations. pre-operative skin markings were made at the superior and inferior poles of the patella to ensure consistent placement of a flexible tape measure across all post-operative time points (24, 48, and 72 h), with the knee in full extension. Three repeated measurements were averaged at each time point.

Post-operative hemoglobin decline was defined as the difference between pre-operative hemoglobin levels and levels measured on the morning of the first post-operative day.

Knee ROM was assessed three times daily at 6-h intervals using a goniometer, with the highest value recorded each day.

All complications occurring during the study period were documented, including nausea, vomiting, post-operative transfusion events, quadriceps weakness, venous thrombotic events, wound complications (e.g., persistent oozing or delayed healing), nerve injury, and falls.

### Sample size estimation

A preliminary pilot study involving 30 patients (not included in the main trial) randomized to either the control or TXA group was conducted to support sample size estimation. The mean VAS pain scores at rest at 24 h post-operatively were 4.1 ± 0.8 in the control group and 3.6 ± 0.6 in the TXA group. Using PASS 2023 software ((NCSS, LLC, Kaysville, UT, USA), a minimum of 44 participants per group was determined to detect this difference with 90% power at a two-sided alpha level of 0.05. Accordingly, the sample size was increased to 50 patients per group to account for potential loss to follow-up.

### Statistical analysis

All analyses were performed using SPSS 26.0 (IBM, Chicago, IL, USA). Data are presented as mean ± standard deviation for continuous variables and as counts (percentages) for categorical variables. Normality was assessed using histograms and quantile–quantile plots. Between-group comparisons were conducted as follows: Student’s *t*-test for normally distributed continuous data; Mann–Whitney U test for skewed or ordinal data (such as total morphine consumption, which was measured in discrete increments and did not follow a normal distribution); Pearson’s chi-square or Fisher’s exact test for categorical variables. For longitudinal data (VAS pain scores at multiple time points), repeated-measures analysis of variance (RM-ANOVA) was employed to assess the effects of time, group, and their interaction. Sphericity was assessed using Mauchly’s test, and the Greenhouse–Geisser correction was applied if violated. Time to first rescue analgesia was analyzed using Kaplan–Meier survival analysis [32, 33], with between-group comparisons performed using the log-rank test. A two-sided *p*-value < 0.05 was considered statistically significant.

## Results

### Patient demographic characteristics

**Fig. 1:**
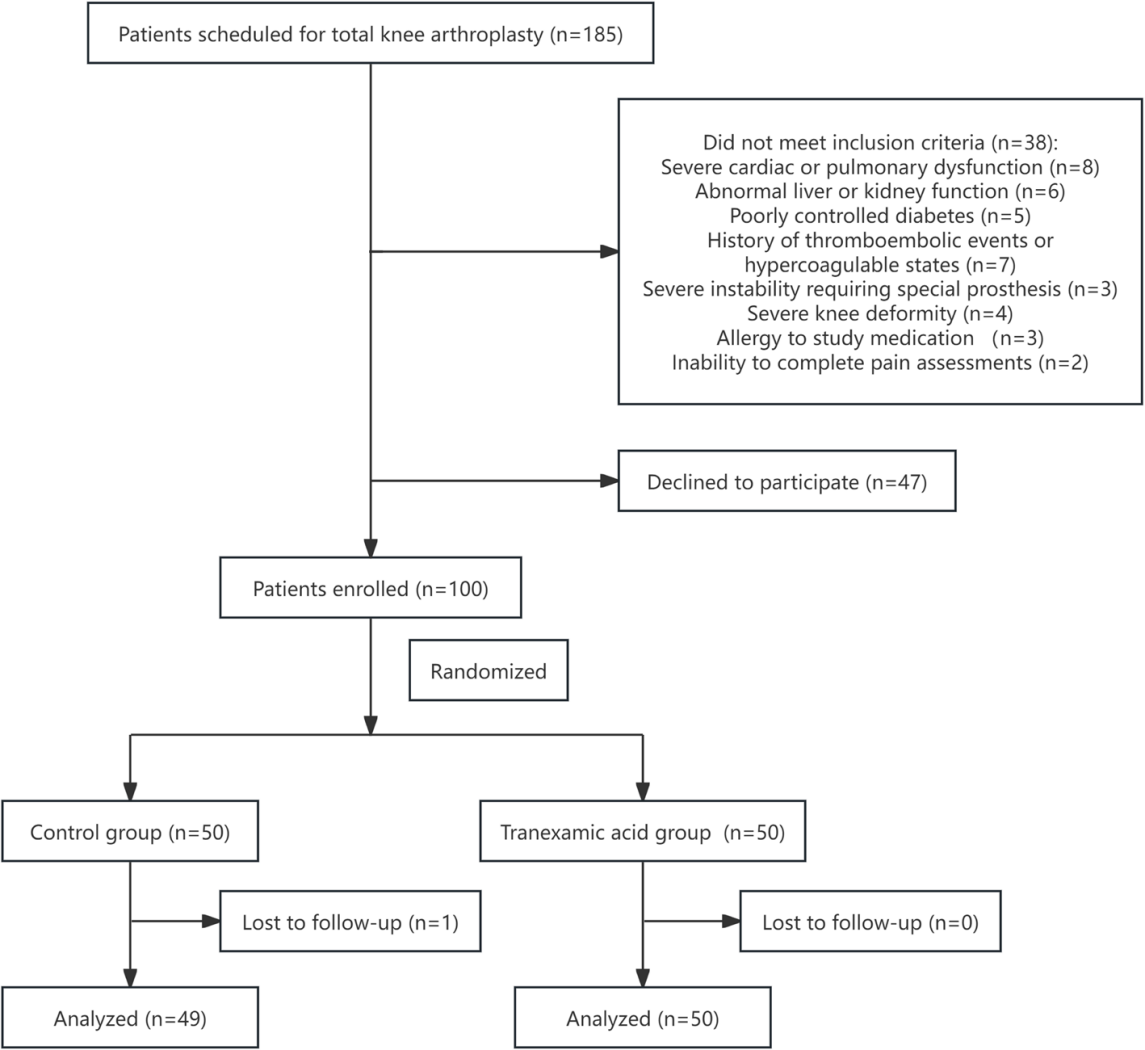
Flow diagram of patient enrollment

Among 185 patients assessed for eligibility, 38 did not meet the inclusion criteria and 47 declined to participate (Fig. 1). A total of 100 patients were ultimately enrolled and randomly assigned to the two groups. During post-operative follow-up, one patient in the control group was excluded due to inadvertent use of analgesic medications outside the study protocol. Consequently, 49 patients in the control group and 50 in the TXA group were included in the final analysis. No significant differences in baseline demographic or clinical characteristics were observed between the two groups before surgery (Table 1).

**Table 1:** Baseline clinicodemographic characteristics of patients

Characteristic	Control group (*n* = 49)		TXA group (*n* = 50)	*p*
Age, yr	66.7 ± 7.5	67.8 ± 6.5		0.434 ^a^
Men/women	18/31	21/29		0.592 ^b^
Weight, kg	68.0 ± 6.6	67.4 ± 10.1		0.701 ^a^
Height, cm	160.9 ± 6.7	160.3 ± 7.6		0.720 ^a^
Body mass index, kg/m^2^	26.3 ± 2.5	26.2 ± 3.1		0.750 ^a^
Surgery side, right/left	27/22	23/27		0.365 ^b^
**Preoperative measures**				
Pain score on visual analog scale	4.7 ± 1.0	4.7 ± 0.9		0.889 ^c^
Range of knee motion, °	109.0 ± 12.9	109.5 ± 14.0		0.694 ^c^
ASA status, I/II/III	0/34/15	0/38/12		0.462 ^c^
Duration of surgery, min	70.3 ± 11.1	72.8 ± 9.5		0.160 ^c^

^a^ Student’s *t*-test; ^b^ Pearson’s chi-squared test; ^c^ Mann–Whitney U test

Values are mean ± standard deviation or *n*, unless otherwise noted

ASA, American Society of Anesthesiologists; TXA, Tranexamic acid

### Primary outcome

**Fig. 2:**
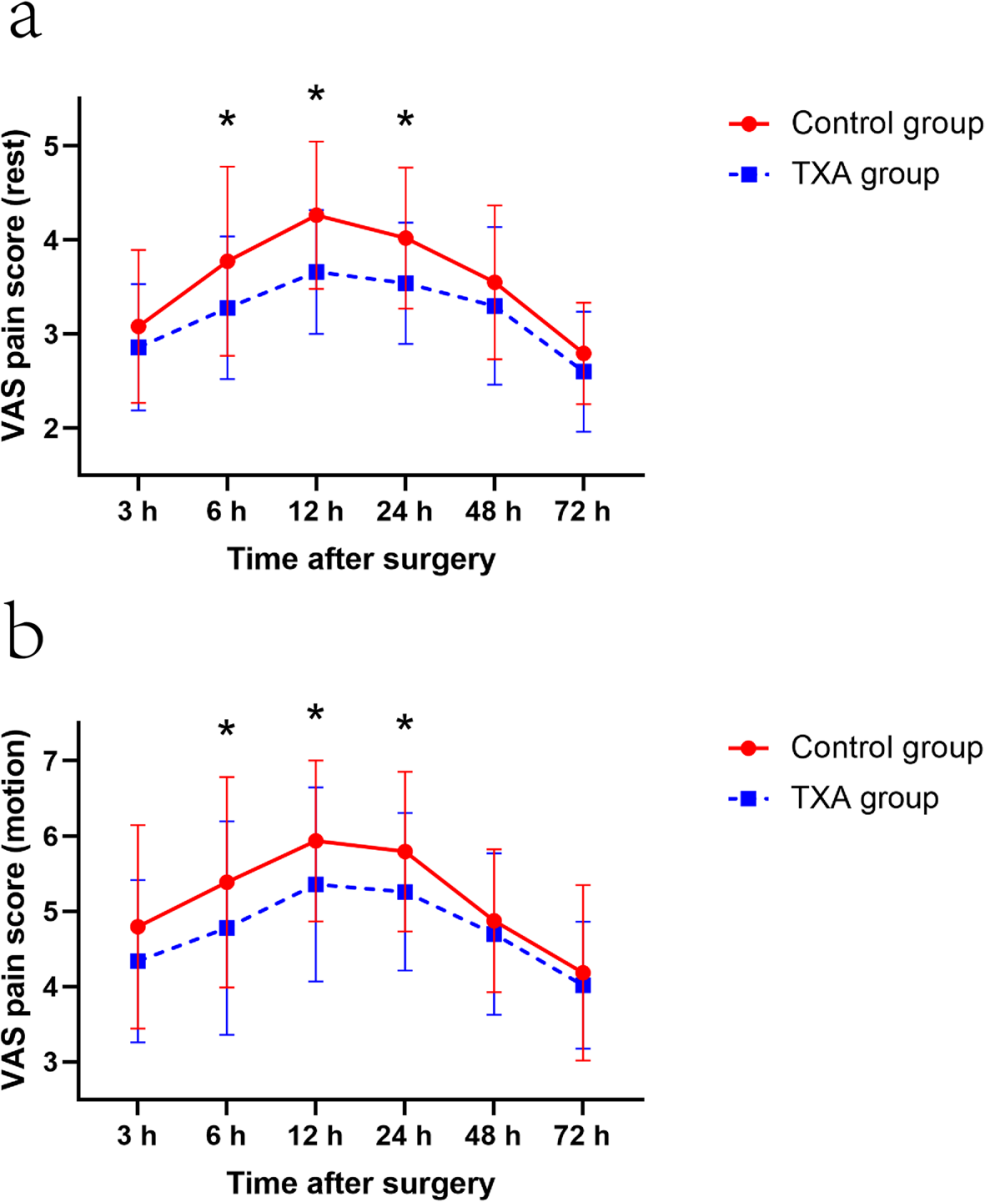
Average pain scores on a visual analogue scale (**a**) at rest or (**b**) during motion at the indicated time points. Data are mean ± SD. *p < 0.05. TXA, tranexamic Acid; VAS, visual analogue scale

Patients in the TXA group demonstrated significantly lower VAS pain scores at rest at 24 h post-operatively compared to the control group (3.5 ± 0.6, 95% CI, 3.4–3.7 vs. 4.0 ± 0.7, 95% CI, 3.8–4.2; *p* = 0.001) (Table 2, Fig. 2). Repeated-measures ANOVA revealed a significant main effect of group for pain at rest (*p* < 0.001), indicating an overall lower pain level in the TXA group across the post-operative period. The time-by-group interaction was not significant (*p *= 0.165). However, the absolute difference in VAS scores at the 24-h time point did not exceed the reported minimal clinically important difference (MCID) for pain in TKA (typically 1.0 point) [34].

**Table 2:** VAS pain scores

Outcome	Control group (*n* = 49)	TXA group (*n* = 50)	Mean Difference (95% CI)	*p*^a^
**VAS pain scores at rest**				
3 h	3.1 ± 0.8	2.9 ± 0.7	0.2 (− 0.1 to 0.5)	0.129
6 h	3.8 ± 1.0	3.3 ± 0.8	0.5 (0.1 to 0.9)	0.011
12 h	4.3 ± 0.8	3.7 ± 0.7	0.6 (0.3 to 0.9)	< 0.001
24 h	4.0 ± 0.7	3.5 ± 0.6	0.5 (0.2 to 0.8)	0.001
48 h	3.6 ± 0.8	3.3 ± 0.8	0.3 (− 0.1 to 0.6)	0.088
72 h	2.8 ± 0.5	2.6 ± 0.6	0.2 (0.0 to 0.4)	0.127
**VAS pain scores on motion**				
3 h	4.8 ± 1.4	4.3 ± 1.1	0.5 (0.0 to 0.9)	0.090
6 h	5.4 ± 1.4	4.8 ± 1.4	0.6 (0.0 to 1.2)	0.034
12 h	5.9 ± 1.1	5.4 ± 1.3	0.6 (0.1 to 1.1)	0.025
24 h	5.8 ± 1.1	5.3 ± 1.0	0.5 (0.1 to 1.0)	0.009
48 h	4.9 ± 0.9	4.7 ± 1.1	0.2 (− 0.2 to 0.6)	0.386
72 h	4.2 ± 1.2	4.0 ± 0.8	0.2 (− 0.2 to 0.6)	0.872

^a^ Mann–Whitney U test

Values are mean ± standard deviation, unless otherwise noted. TXA, Tranexamic acid; VAS, visual analogue scale

### Secondary outcomes

**Fig. 3:**
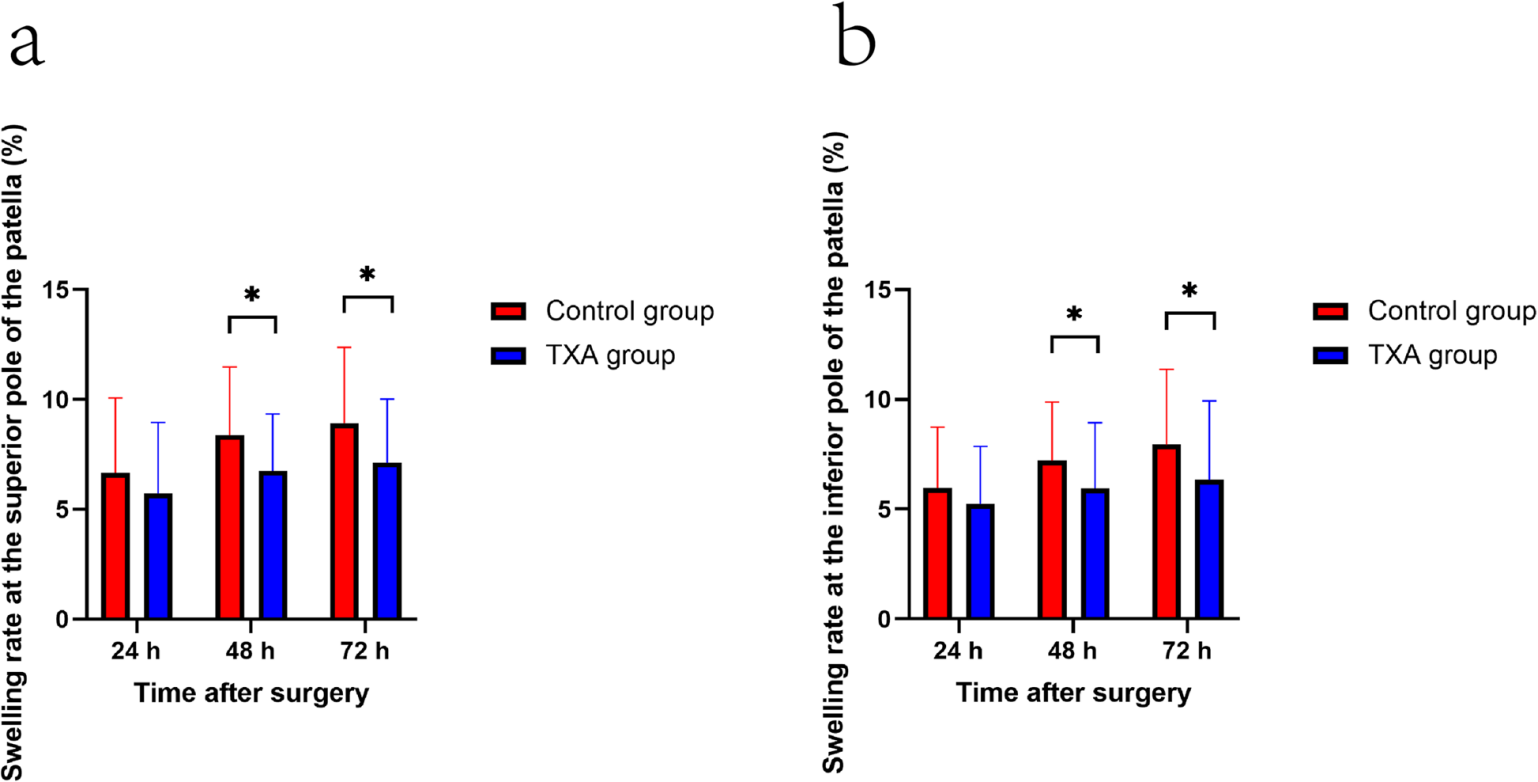
The Knee swelling rate (**a**) at the superior and (**b**) inferior poles of the patella at the indicated time points. Data are mean ± SD. *p < 0.05. TXA, tranexamic Acid

**Fig. 4:**
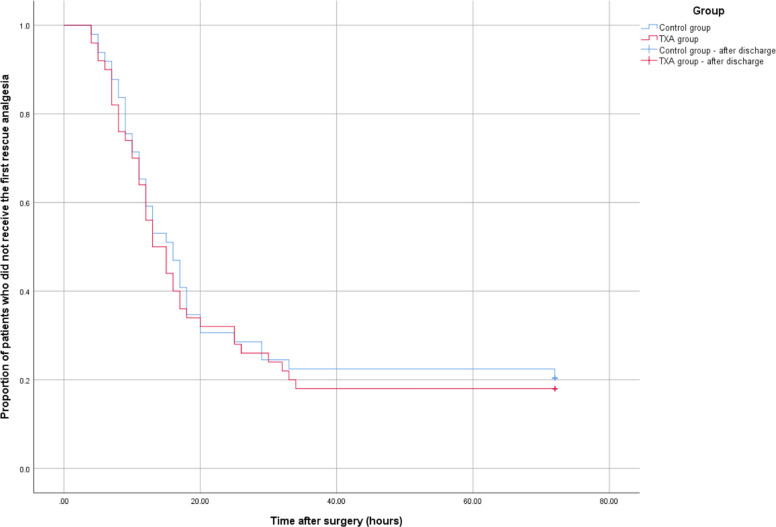
Kaplan–Meier curves of time from the end of surgery until first rescue analgesia with morphine hydrochloride

For VAS pain on motion, repeated-measures ANOVA also showed a significant main effect of group (*p* = 0.001) without a significant time-by-group interaction (*p* = 0.492). Correspondingly, the TXA group exhibited significantly reduced VAS scores at rest at 6 and 12 h and lower VAS scores during motion at 6, 12, and 24 h after surgery compared to controls in post hoc between-group comparisons (Table 2, Fig. 2). However, the absolute differences in VAS scores at these time points also did not exceed the reported MCID. Additionally, knee swelling rates were significantly lower in the TXA group at 48 and 72 h post-operatively (Table 3, Fig. 3). The decline in hemoglobin levels was also markedly less pronounced in the TXA group (33.5 ± 7.5 g/L, 95% CI, 31.4–35.7 vs. 26.2 ± 7.1 g/L, 95% CI, 24.2–28.2; *p* < 0.001). However, no significant intergroup differences were observed in post-operative morphine consumption for rescue analgesia (Table 4), time to first rescue analgesia (Table 4 and Fig. 4), or knee ROM (Table 5).

**Table 3:** The swelling rates of the knee (%)

Outcome	Control group (*n* = 49)	TXA group (*n* = 50)	Mean Difference (95% CI)	*p* ^a^
**Superior pole of the patella**				
24 h	6.7 ± 3.4	5.7 ± 3.2	0.9 (− 0.4 to 2.3)	0.214
48 h	8.4 ± 3.1	6.8 ± 2.6	1.6 (0.5 to 2.8)	0.013
72 h	8.9 ± 3.4	7.1 ± 2.9	1.8 (0.5 to 3.1)	0.011
**Inferior pole of the patella**				
24 h	6.0 ± 2.8	5.2 ± 2.6	0.7 (− 0.4 to 1.8)	0.245
48 h	7.2 ± 2.7	5.9 ± 3.0	1.3 (0.1 to 2.4)	0.025
72 h	7.9 ± 3.4	6.3 ± 3.6	1.6 (0.2 to 3.0)	0.004

^a^ Mann–Whitney U test

Values are mean ± standard deviation, unless otherwise noted

TXA, Tranexamic acid

**Table 4:** Postoperative rescue analgesia

Outcome	Control group (*n* = 49)	TXA group (*n* = 50)	Mean Difference (95% CI)	*p* ^a^
Morphine consumption within 24 h, mg	8.4 ± 5.9	7.8 ± 5.1	0.6 (− 1.6 to 2.8)	0.673 ^a^
Morphine consumption throughout hospitalization, mg	15.5 ± 10.0	13.0 ± 8.4	2.5 (− 1.2 to 6.2)	0.122 ^a^
Time from end of surgery until first rescue analgesia, h	13.7 ± 6.9*	14.1 ± 8.1*	− 0.4 (− 3.8 to 3.0)	0.691 ^b^

Values are mean ± standard deviation, unless otherwise noted

TXA, Tranexamic acid

^a^ Mann–Whitney U test; ^b^ Comparison of Kaplan–Meier curves using the log-rank test based on data from all patients

^*^Based on data from the subset of 38 patients in the control group and 41patients in the TXA group who received rescue analgesia

**Table 5:** Range of knee motion

Outcome	Control group (*n* = 49)	TXA group (*n* = 50)	Mean Difference (95% CI)	*p* ^a^
**Range of knee motion, °**				
Postoperative day 1	95.1 ± 10.4	97.1 ± 11.3	− 2.0 (− 6.3 to 2.3)	0.393
Postoperative day 2	104.3 ± 9.1	106.1 ± 11.6	− 1.8 (− 6.0 to 2.3)	0.226
Postoperative day 3	110.7 ± 7.2	112.0 ± 8.3	− 1.3 (− 4.4 to 1.8)	0.370

^a^ Mann–Whitney U test

TXA, Tranexamic acid

Values are mean ± standard deviation, unless otherwise noted

The incidence of nausea, vomiting, and wound complications during hospitalization was comparable between the two groups (Table 6). None of the patients experienced post-operative transfusion events, significant quadriceps strength reduction, venous thrombotic events, nerve injury, or falls during the study period.

**Table 6:** Postoperative complications

Complication	Control group (*n* = 49)	TXA group (*n* = 50)	Risk Ratio (95% CI)	*p* ^a^
Nausea	9 (18.4)	8 (16.0)	1.15 (0.48 to 2.73)	0.755
Vomiting	4 (8.2)	3 (6.0)	1.36 (0.31 to 5.97)	0.978
Wound complication	3 (6.1)	5 (10.0)	1.22 (0.30 to 4.94)	0.735
Postoperative blood transfusion	0	0		
Decreased quadriceps strength	0	0		
Venous thrombotic event	0	0		
Nerve damage	0	0		
Fall	0	0		

^a^ Pearson’s chi-squared test

Values are *n* (%), unless otherwise noted

## Discussion

This study appears to represent the first RCT evaluating the efficacy and convenience of a TXA-containing analgesic cocktail on post-operative pain, swelling, and blood loss following TKA. The findings from this investigation demonstrate that the addition of TXA to a commonly used LIA cocktail led to a statistically significant reduction in early post-operative pain and swelling, along with decreased blood loss, which may offer added convenience by streamlining the workflow. However, the reduction in pain did not meet the MCID, indicating limited clinical relevance in terms of analgesia.

Topical administration of TXA has been demonstrated to effectively reduce blood loss following TKA [12]. When applied locally, TXA acts directly on the surgical site, achieving high concentrations within the joint cavity, effectively inhibiting excessive fibrinolysis and reducing bleeding in the operative area [35]. Studies suggest that the commonly used dose for topical TXA ranges from 1 to 2 g. Considering that intravenous TXA was also administered in this study, a lower topical dose (1 g) was selected. The classic method for topical TXA delivery is intra-articular injection [23]. However, since both LIA and intra-articular injection of TXA involve the introduction of a large volume of normal saline into the joint cavity, fluid overload may increase intra-articular pressure, exacerbate inflammatory responses, and cause tissue damage or impaired wound healing. Consequently, recent research has proposed peri-articular infiltration of TXA—either via direct injection around the joint or incorporation into the LIA cocktail [36]. Evidence indicates that peri-articular infiltration of TXA is comparable to intra-articular injection in reducing post-operative blood loss and transfusion rates after TKA [36, 37]. Thus, peri-articular infiltration of TXA represents a viable alternative to intra-articular administration.

Although the incorporation of TXA into the LIA cocktail has been proposed in recent years, the majority of existing studies have focused exclusively on its efficacy in reducing blood loss and transfusion rates following TKA [36, 37]. Consistent with previous findings, the results of the present study also indicate that adding TXA to the analgesic cocktail attenuates the post-operative decline in hemoglobin levels, reflecting a reduction in blood loss. However, no prior research has systematically evaluated the effects of TXA-containing cocktails on post-operative pain and swelling after TKA.

Regarding the effect of topical TXA on post-operative swelling after TKA, one study reported no significant impact when TXA was injected directly into the peri-articular tissues [38], while another demonstrated that intra-articular TXA administration could alleviate knee swelling [39]. A recent RCT further indicated that peri-articular injection of TXA combined with intra-articular administration resulted in less soft tissue swelling compared to a combination of intravenous and intra-articular TXA [40]. To date, no study has specifically evaluated the effect of incorporating TXA into the LIA cocktail on post-operative swelling. Our results indicated that adding TXA to the cocktail also reduces early post-operative knee swelling. Previous studies have suggested that swelling on the first post-operative day is generally mild, but becomes more pronounced from the second to third day due to increased mobilization [11, 29]. Our results are consistent with this pattern: while no significant intergroup difference in knee swelling rate was observed on post-operative day 1, the TXA group exhibited significantly lower swelling rates on days 2 and 3 compared to the control group.

Regarding the effect of topical TXA on post-operative pain after TKA, initial studies indicated that topical TXA might even exacerbate pain and opioid consumption following total hip arthroplasty [41]. However, the scenario appears distinct for TKA. Evidence suggests that intra-articular injection of TXA can reduce pain alongside decreasing blood loss [42]. Beyond intra-articular administration, recent research has explored direct peri-articular injection of TXA for TKA. While peri-articular TXA injection may improve knee ROM [43], it demonstrates no significant impact on post-operative pain [43, 44]. Other studies report comparable efficacy between peri-articular and intra-articular TXA injection in reducing both blood loss and pain after TKA [45]. Furthermore, combined intra-articular and peri-articular TXA administration has been associated with attenuated post-operative pain [46]. To our knowledge, no prior study has specifically evaluated the effect of incorporating TXA into the LIA cocktail on post-operative pain after TKA. Our findings indicate that adding TXA to the cocktail results in a statistically significant reduction in early post-operative VAS pain scores. However, the magnitude of these reductions did not reach the threshold of the MCID, and no significant opioid-sparing effect was observed. This suggests that while the addition of TXA to the conventional analgesic cocktail provides a measurable effect, its contribution to overall pain control in this setting is modest. Therefore, further investigations are warranted to explore strategies-such as optimizing the dose or combination of agents-for enhancing the analgesic efficacy of TXA-containing LIA formulations.

The high inter-patient variability in post-operative morphine consumption is a notable finding. This underscores the individualized nature of pain and opioid requirement after TKA, which may be influenced by differences in pain sensitivity, anxiety, pre-operative opioid exposure, and motivation during rehabilitation. The lack of a significant between-group difference in morphine use, despite lower pain scores in the TXA group, suggests that the observed pain reduction-while statistically significant-was not of a magnitude sufficient to meaningfully alter rescue analgesia needs within the context of this variability and the background of effective multimodal analgesia.

Significantly enhancing the analgesic efficacy of LIA remains challenging due to the absence of a gold-standard formulation, with considerable variation in adjuvant use across studies and institutions [47–49]. A recent network meta-analysis of 43 RCTs confirmed that different adjuvant combinations offer distinct advantages in reducing pain scores, or decreasing opioid consumption, yet an optimal regimen remains elusive [50]. In this context, and based on institutional experience and prior reports [16, 48], we added TXA to a conventional cocktail. Identifying an optimal formulation remains an important goal and necessitates further investigation.

This study has several limitations. First, the assessment of patient-centered and functional outcomes was limited. The study focused on the early post-operative period and did not include validated, osteoarthritis-specific patient-reported outcome measures (PROMs) such as the WOMAC or Oxford Knee Score, which restricts the evaluation of recovery beyond hospitalization. Furthermore, we did not quantitatively assess pre-operative physical activity levels or post-operative mobilization adherence. Although all patients followed a standardized rehabilitation protocol to minimize variability in post-operative activity, the lack of this baseline and process data represents a limitation in fully contextualizing the recovery outcomes. Second, the sample size calculation was based on the VAS score at rest at 24 h post-operatively—a commonly used primary outcome in arthroplasty analgesia studies [50]. Consequently, the study may have been underpowered to detect differences in other secondary outcome measures. Third, the method of allocation concealment utilized sequentially numbered, opaque, sealed envelopes. While this method was implemented with strict procedures (sequential opening by a non-blinded investigator), it is recognized to have a higher risk of bias compared to centralized, web-based randomization systems. This is a limitation of our single-center study design. Fourth, as previously noted, the findings originate from a single-center setting. Therefore, future multicenter studies with extended follow-up durations and varied multimodal analgesia protocols are warranted to validate these results. Furthermore, knee swelling was measured using a flexible tape, a method subject to inherent variability. Despite standardization and averaging of triplicate measurements by a single blinded assessor, we did not formally evaluate its reliability (e.g., via Intra-class Correlation Coefficient), which is a methodological limitation. Future studies may benefit from more objective modalities like ultrasound or MRI.

In conclusion, the addition of TXA to a commonly used LIA cocktail (ropivacaine + epinephrine + dexamethasone) led to a statistically significant reduction in early post-operative pain and swelling, along with decreased blood loss, which may offer added convenience by streamlining the workflow. However, the reduction in pain did not meet the MCID, indicating limited clinical relevance in terms of analgesia. Future studies are needed to optimize the analgesic efficacy of TXA-containing cocktails.

## Supplementary Information

Supplementary Material 1.

## Data Availability

The datasets used and/or analyzed during the current study are available from the corresponding author on reasonable request.
