# Supplementary material for: Tranexamic acid in local infiltration analgesia cocktail for pain and swelling after total knee arthroplasty: a randomized controlled trial
Source: Arthroplasty. 2026 Apr 13;8:28. doi: 10.1186/s42836-026-00385-8 (PMC13072563; doi:10.1186/s42836-026-00385-8)
Supplement: Supplementary file 1 — Supplementary Material 1. [file 42836_2026_385_MOESM1_ESM.docx]

**Patient Inclusion and Exclusion Criteria**

We consecutively enrolled patients meeting the following criteria: (1) aged 18-80 years; (2) scheduled for unilateral primary TKA for knee osteoarthritis; (3) admitted to our institution between August 2024 and June 2025; (4) having an American Society of Anesthesiologists (ASA) physical status classification of I to III.

Exclusion criteria comprised: (1) Severe cardiac or pulmonary dysfunction; (2) Abnormal liver or kidney function (e.g., ALT or AST > 1.5 times the upper limit of normal; BUN > 8.3 mmol/L; Scr > 115 μmol/L); (3) Poorly controlled diabetes with infection risks as judged by the investigators; (4) Impaired consciousness or psychiatric disorders; (5) Previous open knee surgery; (6) Neuromuscular disorders affecting lower limb function; (7) A history of thromboembolic events (e.g., deep vein thrombosis, pulmonary embolism, ischemic stroke) or known hypercoagulable states; (8) severe bleeding disorders; (9) Comorbidities rendering the patient unfit for surgery; (10) Severe instability requiring ligament-retaining TKA; (11) Active systemic or local infections; (12) Severe osteoporosis, metabolic bone disease, radiation-induced bone pathology, or tumors around the knee; (13) Pregnancy, lactation, or planned pregnancy during the study period; (14) Participation in another clinical trial within the past three months; (15) Other contraindications deemed significant by the investigators; (16) Knee flexion deformity ≥ 30° or varus/valgus deformity ≥ 30°; (17) Allergy to any study medication; (18) Opioid dependence; (19) Inability to complete pain assessments due to language or cognitive barriers.
